# Fungi stabilize multi‐kingdom community in a high elevation timberline ecosystem

**DOI:** 10.1002/imt2.49

**Published:** 2022-08-15

**Authors:** Teng Yang, Leho Tedersoo, Xu Liu, Gui‐Feng Gao, Ke Dong, Jonathan M. Adams, Haiyan Chu

**Affiliations:** ^1^ State Key Laboratory of Soil and Sustainable Agriculture, Institute of Soil Science Chinese Academy of Sciences Nanjing China; ^2^ University of Chinese Academy of Sciences Beijing China; ^3^ Mycology and Microbiology Center University of Tartu Tartu Estonia; ^4^ Life Science Major Kyonggi University Suwon South Korea; ^5^ School of Geographic and Oceanographic Sciences Nanjing University Nanjing China

**Keywords:** connectivity, fungi, multiple‐kingdom networks, modularity, stability, timberline ecosystems

## Abstract

Microbes dominate terrestrial ecosystems via their great species diversity and vital ecosystem functions, such as biogeochemical cycling and mycorrhizal symbiosis. Fungi and other organisms form diverse association networks. However, the roles of species belonging to different kingdoms in multi‐kingdom community networks have remained largely elusive. In light of the integrative microbiome initiative, we inferred multiple‐kingdom biotic associations from high elevation timberline soils using the SPIEC‐EASI method. Biotic interactions among plants, nematodes, fungi, bacteria, and archaea were surveyed at the community and network levels. Compared to single‐kingdom networks, multi‐kingdom networks and their associations increased the within‐kingdom and cross‐kingdom edge numbers by 1012 and 10,772, respectively, as well as mean connectivity and negative edge proportion by 15.2 and 0.8%, respectively. Fungal involvement increased network stability (i.e., resistance to node loss) and connectivity, but reduced modularity, when compared with those in the single‐kingdom networks of plants, nematodes, bacteria, and archaea. In the entire multi‐kingdom network, fungal nodes were characterized by significantly higher degree and betweenness than bacteria. Fungi more often played the role of connector, linking different modules. Consistently, structural equation modeling and multiple regression on matrices corroborated the “bridge” role of fungi at the community level, linking plants and other soil biota. Overall, our findings suggest that fungi can stabilize the self‐organization process of multi‐kingdom networks. The findings facilitate the initiation and carrying out of multi‐kingdom community studies in natural ecosystems to reveal the complex above‐ and belowground linkages.

## Highlights

- Multi‐kingdom network construction increased network complexity.
- Multi‐kingdom network construction increased negative edge proportion.
- Fungi stabilized multi‐kingdom network structure.
- Fungi linked above‐ and below‐ground biota at the community level.

## Graphical abstract

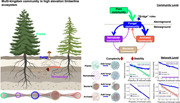

## INTRODUCTION

Among all terrestrial ecosystems, forests constitute the largest carbon (C) sink, slowing the continuous increase in atmospheric carbon dioxide concentrations, with implications for global climate change [1]. For example, the global forest ecosystems reportedly sequestered 21.5 Pg C in 2001–2010 [2]. In addition, forests play important roles, such as wood production and water, soil, and biodiversity conservation. Human activities and climate change pose threats to forest ecosystems through deforestation, wildfire, drought, as well as disease and insect pest outbreaks [3]. The responses of forest ecosystems to such general disturbances are influenced considerably by biotic interactions among plants and soil biota [4]. A comprehensive investigation of the characteristics and structures of the hyper‐diverse belowground biota in forests is essential, considering belowground biodiversity actively shapes aboveground biodiversity and biogeochemical processes [5, 6].

Network theory and its application have greatly enhanced our understanding of various biotic interactions in complex systems [7, 8]. Network analyses not only model the general co‐occurrence patterns of microbes and hosts [9, 10] but also disentangle the microbe–microbe and microbe–host interactions [11, 12]. In such cases, the keystone species (e.g., connectors, module hubs, and network hubs [7, 13]) and major modules [14, 15] essential for community assembly and ecosystem functioning are determined, and the significant influences of biotic interactions on system stability or host health are deduced [16].

Recently, the concept of “integrative microbiome” has been proposed as a direction for future microbiome studies, to include all protists, fungi, bacteria, archaea, and viruses [17]. Such multiple‐kingdom network analyses can reveal the co‐occurrence patterns of different microbial kingdoms in the same area as well as the respective roles of different taxonomic groups in multi‐kingdom communities. In the Tibetan plateau, we observed that archaea play a critical role in constructing soil microbial co‐occurrence networks; the omission of the archaeal community resulted in a remarkable decline in natural connectivity in the entire network [18]. Similarly, fungi have been observed to play a pivotal role in stabilizing association networks in some environments, such as in human lung and skin systems [19], and in a grassland under drought stress [20].

High elevation timberline ecosystems are characterized by a unique temperature‐limited upper elevational boundary of closed forests that is highly sensitive to climate change and human activity [21, 22]. In most cases, the upper elevational boundary is not a clear line. Instead, it is often a broad ecotone between closed forests and alpine grasslands or tundra. In addition, one or two timberline tree species that cover the entire high elevation timberline ecosystem usually inhabit the ecotone. Ectomycorrhizal (EcM) fungi are mutualistic with timberline trees across temperate and boreal mountains; they enhance water [23] and nutrient uptake (mainly nitrogen [N] [24]) in host plants and thus broaden plant distributional ranges [25]. In addition to the underground EcM fungi [26], there are other abundant belowground or soil surface fungi (hereafter referred to as non‐EcM fungi) in the timberline, such as saprotrophic fungi in leaf litter [27], ericoid\arbuscular mycorrhizal fungi (AMF), and non‐mycorrhizal endophytic fungi at the root–soil interface [28, 29, 30]. Such diverse fungal community jointly constitutes a complex and advanced fungal network in belowground ecosystems [31], which drives plant population and community as well as soil nutrient dynamics [32, 33]. Despite the vital roles of the belowground fungal community, our understanding of the relationships between the underground fungal community and other soil biota, especially in timberline ecosystems, remains poor.

The Erman's birch forest on Changbai Mountain is one of the most well‐protected alpine forests in northeast Asia. The timberline tree species, *Betula ermanii* Cham., covers a ca. 450‐m vertical range in the upper part of the mountain forest [34]. In the present study, we surveyed the multi‐kingdom community, including nematodes, fungi, bacteria, and archaea, in neighboring soils of a single tree species (*B. ermanii*) across its native range on Changbai Mountain, China. We constructed the multi‐kingdom association network (including plants, nematodes, fungi, bacteria, and archaea) and examined the respective roles of taxa belonging to different kingdoms in the entire network. We hypothesized that (1) multi‐kingdom network construction would largely increase the complexity and negative edge proportion (negative edge number/total edge number) when compared with that in single‐kingdom networks because introducing more species from different kingdoms is assumed to add to trophic complexity and inter‐specific competition for available nutrients, particularly under harsh environments. For example, introducing nematodes will add prey‐predator relationships, such as plant feeder, hyphal feeder, and bacterial feeder [35], which will increase trophic complexity in food webs. In forests, the presence of EcM fungi can help plants to compete with soil free‐living microbes for limited N [36]. This high‐order interaction, namely, that the presence of a species influences the interaction between other species, has been proven to stabilize the competitive network models [37]. (2) Considering the predominance of EcM networks and high fungal biomass in timberline ecosystems, species belonging to the fungal kingdom are crucial for the stability of multi‐kingdom community association networks. In addition, EcM fungi are more important for stabilizing multi‐kingdom networks than non‐EcM fungi. (3) Considering the reported associations in community composition between fungi and other biota (including plants) [38, 39, 40], the fungal community would be the “bridge” of the multi‐kingdom community, namely, fungal community composition would significantly affect the communities of plants and other soil biota, and vice versa.

## RESULTS

### Comparison of multi‐ and single‐kingdom networks

**Figure 1:**
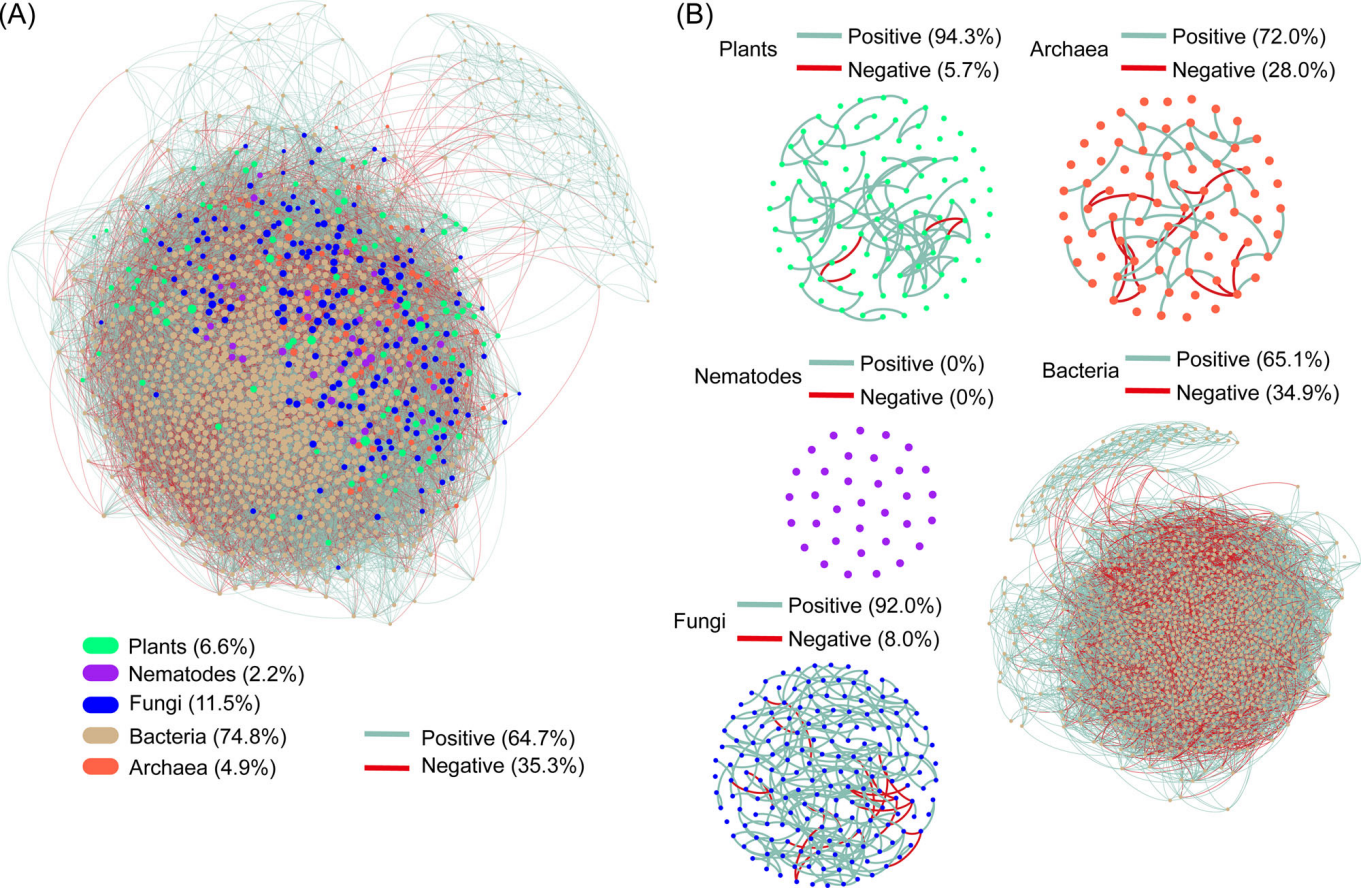
The multi‐kingdom (A) and single‐kingdom (B) association networks in the timberline ecosystem of Changbai Mountain. The single‐kingdom network of nematodes did not have any edges.

**Figure 2:**
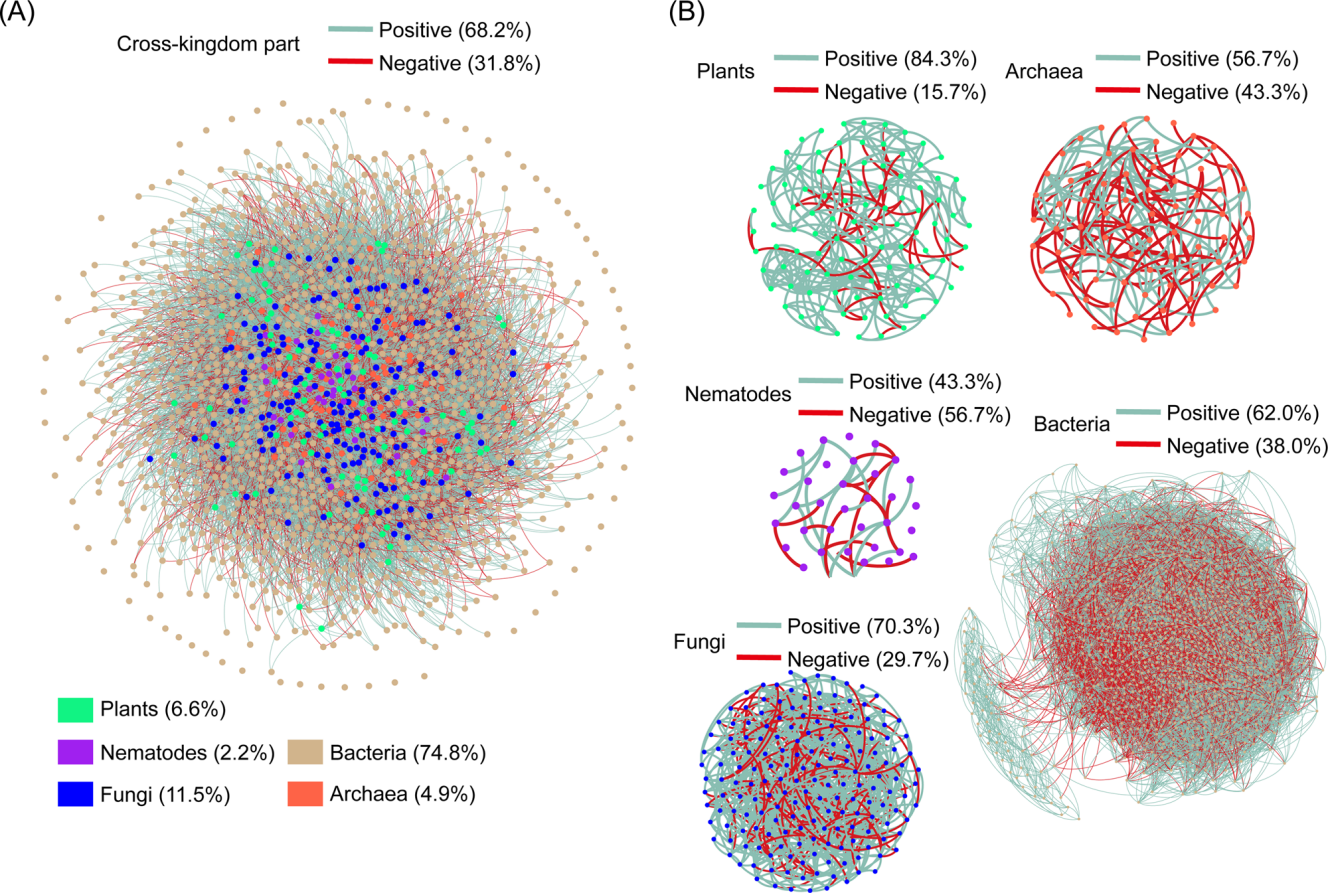
Disassembly of the entire multi‐kingdom association network. (A) The cross‐kingdom subset showing all the cross‐kingdom edges in the entire multi‐kingdom network, such as plant‐fungus, fungus‐bacterium, and bacterium‐archaea links. (B) The five within‐kingdom subsets showing all the within‐kingdom edges in the entire multi‐kingdom networks, including plant‐plant, nematode‐nematode, fungus‐fungus, bacterium‐bacterium, and archaea‐archaea links.

By using multi‐marker metabarcoding, we constructed multi‐ and single‐kingdom networks of plants, nematodes, fungi, bacteria, and archaea, separately (Figure 1). There were 88, 0, 286, 15,824, and 50 edges for the single‐kingdom networks of plants, nematodes, fungi, bacteria, and archaea, respectively. The multi‐kingdom network contained 1550 nodes and 28,032 edges, including 38.4% of cross‐kingdom edges and 35.3% of negative edges. Among the single‐kingdom networks, bacterial and fungal networks had the largest node number, edge number, and mean connectivity; however, they had the lowest modularity (Table 1). The plant and fungal networks were characterized by low proportions of negative edges (<10%), while bacterial and archaeal networks had 34.9% and 28.0% of negative edges, respectively (Figure 1B). Compared to those in the single‐kingdom networks, the edge number and mean connectivity were enhanced by 1012 and 0.6, respectively, in the within‐kingdom subsets of the multiple‐kingdom network, and the negative edge proportion increased by 2.9% (Table 2, Figures 1 and 2). In particular, the increases in mean connectivity and negative edge proportion were the most significant for the fungal subset: the mean connectivity increased from 3.2 to 8.7, and the negative edge proportion increased from 8.0% to 29.7% (Table 2, Figures 1B and 2B).

**Table 1:** Topological characteristics of multi‐ and single‐kingdom networks

Network type	Node Number	Edge Number	Mean connectivity	Modularity	Average path length
Multi‐kingdom	1550	28,032	36.2	0.182	2.51
Plants	102	88	1.7	0.840	2.46
Nematodes	34	0	0	0	0
Fungi	178	286	3.2	0.695	5.81
Bacteria	1160	15,824	27.3	0.208	2.67
Archaea	76	50	1.3	0.847	2.46

**Table 2:** Differences in topological characteristics between single‐kingdom networks and the within‐kingdom subsets of the multi‐kingdom network

Network type	Node Number	Edge Number	Mean connectivity	Modularity	Average path length
*Single‐kingdom networks*					
Plants	102	88	1.7	0.840	2.46
Nematodes	34	0	0	0	0
Fungi	178	286	3.2	0.695	5.81
Bacteria	1160	15824	27.3	0.208	2.67
Archaea	76	50	1.3	0.847	2.46
*Within‐kingdom subsets of multi‐kingdom network*					
Plants	102	262	5.1	0.615	3.62
Nematodes	34	30	1.8	0.595	3.57
Fungi	178	771	8.7	0.355	2.69
Bacteria	1160	16010	27.6	0.212	2.63
Archaea	76	187	4.9	0.436	2.98

**Figure 3:**
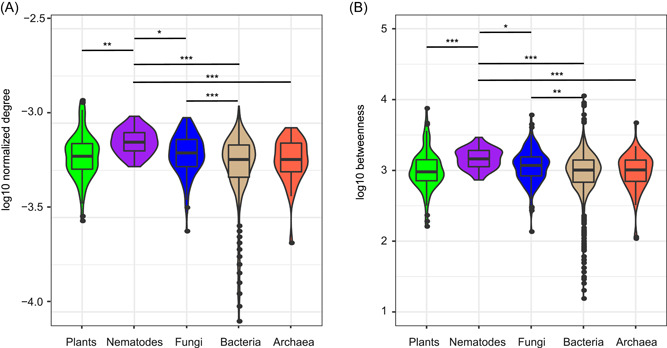
Node connectedness and centrality of plants, nematodes, fungi, bacteria, and archaea in the multi‐kingdom network. (A) Node connectedness is represented by log10 normalized degree, and (B) centrality is represented by log10 betweenness. The results of post‐hoc Kruskal–Wallis test are shown in diagrams. *<0.05, **<0.01, and ***<0.001.

In the multi‐kingdom network, fungi and bacteria were the two largest kingdoms in terms of node number, edge number, and mean connectivity (Table 2). At the node level, the normalized degree and betweenness of fungi were significantly larger than those of bacteria, and the normalized degree and betweenness of nematodes were the highest among all the groups (Figure 3). Normalized degree and betweenness were not significantly different between EcM and non‐EcM fungi (Figure S1). The *z*‐*c* plot showed the relative roles of plants, nematodes, fungi, bacteria, and archaea in the multiple‐kingdom network structure (Figure S2). Specifically, there were 13, 4, 41, 296, and 27 connectors for plants, nematodes, fungi, bacteria, and archaea, respectively. The taxonomic and functional affinities of 41 fungal connectors are summarized in Table S1. Twenty out of the 41 fungal connectors were species of EcM fungi, including *Cortinarius* (9 nodes), *Russula* (4 nodes), and *Tomentella* (4 nodes). In addition, seven fungal connectors belonged to the saprotrophic mold genus, *Mortierella*, and five nodes belonged to the AMF phylum *Glomeromycota*. Among other organisms, there were two network hubs: one archaeal (uncultured Thermoplasmata) and one bacterial (*Geobacter* sp.), and one archaeal module hub (uncultured Nitrososphaeria); plant and nematode species did not act as network or module hubs.

### Roles of fungi and other kingdoms in multi‐kingdom networks

**Figure 4:**
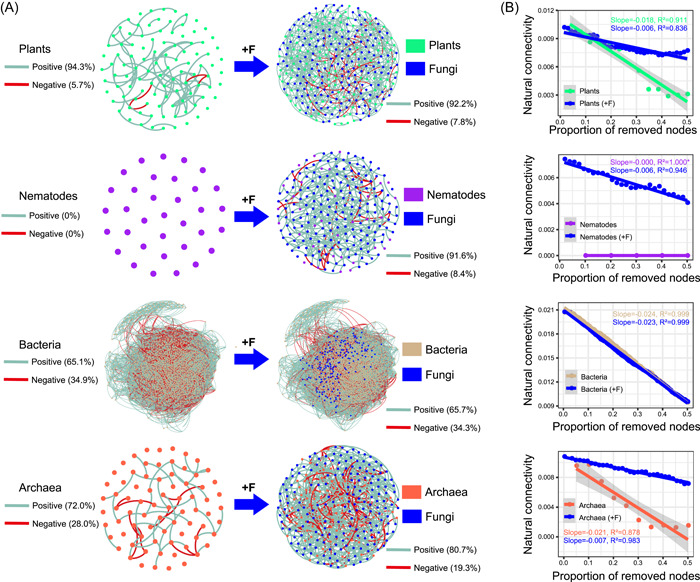
The additive impacts of fungal interactions on single‐kingdom networks of plants, nematodes, bacteria, and archaea. (A) Changes in network topology with fungal interaction addition. (B) Changes in network stability with fungal interactions addition. The decreasing trend of natural connectivity is fitted with 50% nodes lost, and the *R* ^2^ and slope are shown in diagrams. The lower the absolute value of the slope, the more stable the network. “+F” represents the addition of fungal interactions on the basis of single‐kingdom networks.

We examined the stability of association networks in the presence and absence of fungi and other kingdoms. Following the addition of fungal interactions into the single‐kingdom networks of plants, nematodes, bacteria, and archaea, the networks became more connected and less modular (Figure 4A, Table S2). The addition of a bacterial network had effects similar to those of the addition of a fungal network; however, neither nematodes nor archaea had similar effects (Tables S3–S5). In particular, the addition of fungi enhanced the network stability of plants, nematodes, and archaea substantially (Figure 4B). For example, the natural connectivity of single‐kingdom networks of plants and archaea decreased, with slopes of −0.018 and −0.021, respectively, whereas the slopes were elevated to −0.006 and −0.007, respectively, following fungal network addition. Fungal network stability was not enhanced following the addition of nematodes, bacteria, and archaea into the fungal network (Figure S3). Furthermore, the addition of EcM and non‐EcM fungi, separately, enhanced the network stability of plants, nematodes, and archaea (Figure S4). The effects of the addition of EcM and non‐EcM fungi on network topological properties were similar (Table S6).

Plant–fungus networks had remarkably large positive edge proportions (>92.2%), implying the dominance of coexistence rather than mutual exclusion between plants and fungi in timberline ecosystems. Among all the single‐kingdom networks, the lowest absolute value of natural connectivity slope was observed in the fungal network (slope = −0.005; Figure S5A). When the fungal network was removed from the entire multi‐kingdom network, natural connectivity (Figure S5B), edge number, and mean connectivity decreased; however, the modularity increased (Table S2). Only plant, fungal, and archaeal kingdom network removal decreased the stability of the entire multiple‐kingdom network, and fungal removal led to the lowest natural connectivity (Figure S5B). There were no obvious differences in network stability between different fungal guilds in both the individual networks of EcM and non‐EcM fungi or within the entire multi‐kingdom network, excluding EcM and non‐EcM fungi; the only difference might be that EcM fungal network could be more connected compared with non‐EcM fungi (Figure S6).

**Figure 5:**
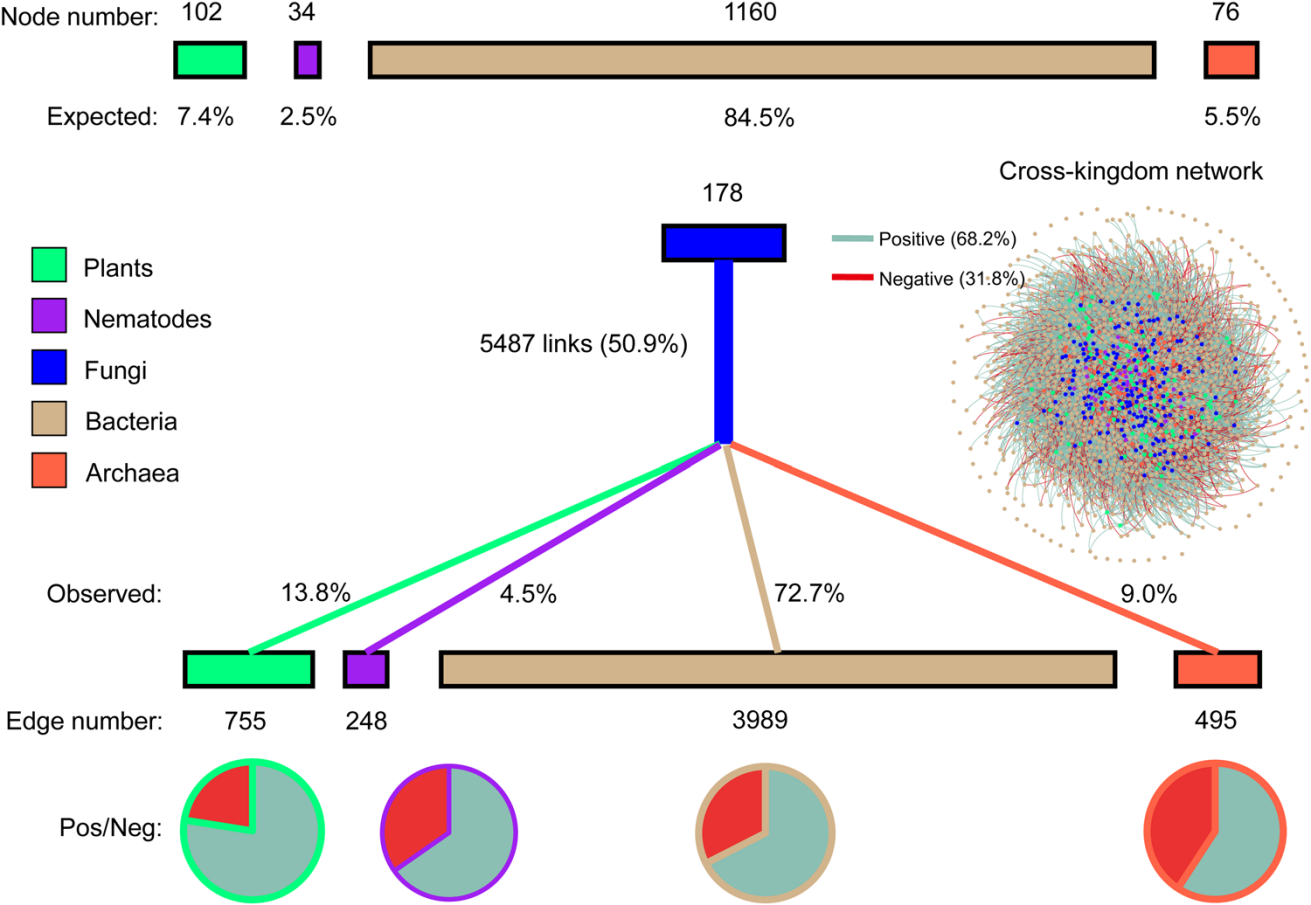
Fungal links in the cross‐kingdom part of the network. The expected proportions of fungal links were based on the node numbers of plants, nematodes, bacteria, and archaea. The observed proportions of fungal links are calculated based on the edge number. Positive (cyan) and negative (brick red) edge proportions are shown using pie plots at the bottom of the diagram.

There were 5487 links between fungi and other kingdoms, which accounted for 50.9% of the total cross‐kingdom links (Figure 5). Based on the node numbers of plants and other soil biota, the expected proportions of fungal links were 7.4%, 2.5%, 84.5%, and 5.5% for plants, nematodes, bacteria, and archaea, respectively. However, the observed proportions of fungal links were 13.8%, 4.5%, 72.7%, and 9.0% for plants, nematodes, bacteria, and archaea, respectively (Figure 5). The positive edge proportions for the fungal links with plants and archaea were the highest (77.5%) and lowest (59.2%), respectively (Figure 5). In addition, significantly higher negative edge proportions were observed in the links of saprotrophic and EcM fungi than in the remaining links within fungi, based on the null model comparison (Figure S7).

### Associations among fungi and other kingdoms at the community level

Considering the 40 noncollinear environmental variables (Figure S8), as well as geographic distance and neighboring plant community as the candidate predictors, we constructed the conventional multiple regression on matrices (called MRM #1) to determine the predictors of nematode, fungi, bacteria, and archaea community composition (Table S7). Soil pH was the strongest predictor of nematode community composition, explaining 20.1% of the variation in community composition. Fungal community composition was mainly influenced by plant community composition, soil pH, and conductivity. Soil pH and available Mg jointly explained 17.3% of the variation in bacterial community composition. Archaeal community composition was mainly affected by tree richness and soil total Fe concentration. When the community composition of soil biota was added, the full MRM (referred to as MRM #2) explained a greater variation in community compositions of nematodes, fungi, and archaea (Table S8). The fungal community strongly affected nematode and archaea community composition (*p* = 0.001); in particular, fungal community composition was the strongest predictor of archaeal community composition (Table S8). Coincidentally, the nematode and archaea community compositions also significantly affected fungal community composition, and plant community composition was still the strongest predictor of fungal community composition (Table S8).

**Figure 6:**
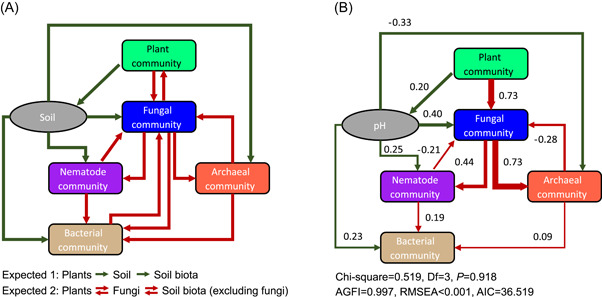
Multi‐kingdom biotic interactions at the community level revealed by structural equation modeling (SEM). (A) The expected model is constructed based on two hypotheses: one is that plant community affects soil biota (incl. fungi) by modifying soil properties; the other is that fungi community affects plants and soil biota (excluding fungi), and vice versa. (B) The best model was selected based on Akaike Information Criterion (AIC), and only significant paths were retained. Line width of each path fitted the size of the standardized path coefficient (SPC) that is shown near each path. The paths of expected #1 are colored dark green, and the paths of expected #2 are colored dark red. In the model, Bray–Curtis dissimilarities were used to represent communities of soil biota, and Jaccard dissimilarities were used to represent plant community. AGFI, adjusted goodness of fit index; AIC, Akaike information criterion; RMSEA, root mean square error of approximation. *N* = 30

Structural equation modeling (SEM) results revealed similar patterns, whereby plant community composition strongly and directly affected fungal community composition, and indirectly affected the compositions of other soil communities through its effects on fungal community composition (Figure 6). Based on the standardized path coefficients (SPC), the fungal community was mostly associated with the plant community and archaeal community (SPC = 0.73, respectively). In addition, the plant community directly affected soil pH, and indirectly affected the communities of nematode, fungi, bacteria, and archaea through its effect on pH (Figure 6).

## DISCUSSION

According to the results of the network analyses in the present study, fungi and bacteria were the two largest biological groups and had the highest node and edge numbers (Figure 1). Nevertheless, there were quite distinct topologies of single‐kingdom networks between bacteria and fungi: the negative edge proportion of the bacterial network was fourfold higher than that of fungi, and the mean connectivity of the bacterial network was about eightfold higher than that of fungi (Figure 1B, Table 1). The results imply that bacteria have a high capacity for self‐organization but compete with each other intensely, whereas fungus‐to‐fungus relationships tend to be more positive and cooperative.

Consistent with our first hypothesis, the within‐kingdom edge numbers, mean connectivity, and negative edge proportion increased dramatically in the multi‐kingdom network when compared to in the single‐kingdom networks (Table 2, Figures 1B and 2B). In particular, fungal mean connectivity increased three‐fold, and its negative edge proportion increased by about fourfold in the within‐kingdom subset (Table 2). The increase in mean connectivity represents network complexity enhancement [15] when other trophic groups are added. The pattern is consistent with the widely held paradigm that diversity is positively correlated with network complexity [41, 42]. The increase in negative edge proportion indicates the intensification of interspecific competition when compared with those in the single‐kingdom networks [10].

N is one of the limiting nutrients considered to strongly affect soil microbial growth and diversity at the treeline [43, 44]. Notably, in both the multi‐ and single‐kingdom networks, the negative edge proportion was significantly higher than expected for the links between saprotrophic and EcM fungi than in the remaining links within the fungal kingdom (Supporting Information: Figure S7). This may be related to the “Gadgil effect,” according to which EcM fungi directly obtain N from organic compounds, leading to N limitation for the free‐living saprobes [45]. Two prokaryote keystone species, the ammonia oxidizer *Nitrososphaeria* (Module hub) and N‐fixing *Geobacter* (Network hub) (Figure S2) were highly correlated with forest N cycling [14, 46]. The findings gave us a glimpse of the tight relationship between microbial network topology and soil biogeochemical cycling. Soil microorganisms, either living or dead, which play pivotal roles in biogeochemical processes, are also likely to be the keystone species in multi‐kingdom communities [47, 48]. Previously, we observed that soil microbial functional diversity (particularly for C and N cycling‐related genes) increased dramatically at the treeline of an ecotone in Changbai Mountain [49].

Consistent with our second hypothesis, the fungal kingdom strongly influenced network connectivity and stability in the multi‐kingdom community. At the node level, the normalized degree and betweenness of fungi were significantly larger than those of bacteria (Figure 3). A higher node degree implies more links from one fungus to other amplicon sequence variants (ASVs), while higher betweenness values indicate that fungi are more likely to be key “brokers” [15]. Previously, fungi have been found to have higher betweenness and node degree than bacteria in bacterial–fungal networks in human lung and skin systems [19]. In the entire multi‐kingdom network in the present study, fungal within‐kingdom links were only 771, whereas fungal cross‐kingdom links reached 5487 (sevenfold higher than within‐kingdom links). Therefore, the importance of fungi was mainly attributable to cross‐kingdom links rather than within‐kingdom links. In addition, in the present study, the 5487 links between fungi and other kingdoms accounted for 50.9% of the total cross‐kingdom links (Figure 5). Compared to the randomized links of fungi, fungi were more prone to linking with plants (+6.4%), archaea (+3.5%), and nematodes (+2.0%). Here, the highest proportion of positive edges (77.5%) was in the fungal links with plants, whereas the highest proportion of negative edges (40.8%) was in the fungal links with archaea (Figure 5). Previously, strong positive relationships in diversity and community composition have been reported between fungi and plants [38], and fungal community dissimilarity significantly increased with an increase in plant phylogenetic distance [50]. The results of the present study further corroborated the strong positive associations between fungal and plant species at the network level. The observed associations between fungi and archaea may result from their different niche preferences for oxic and anoxic environments.

Another notable effect of the fungal kingdom on the multi‐kingdom network was the “stabilizer” effect. When fungal interactions were integrated into the single‐kingdom networks of plants, nematodes, bacteria, or archaea, the networks became more connected and less modular (Figure 4A, Table S2). The addition of fungi enhanced the network stability of plants, nematodes, and archaea substantially (Figure 4B). Notably, the single‐kingdom fungi network was very stable; the absolute value of natural connectivity slope in the fungal network was only 0.005, compared to 0.018, 0.024, and 0.021 for the plant, bacterial, and archaeal single‐kingdom networks, respectively (Figure S5A). Fungal network stability may provide the “basic skeleton” that supports the stability of the entire multi‐kingdom network. Previously, higher network stability for fungi compared to bacteria network stability has also been reported in drought‐stressed grassland mesocosms [20]. When the fungal network was removed from the entire multi‐kingdom network, the natural connectivity decreased overall (Supporting Information: Figure S5B); furthermore, the mean connectivity and edge number were largely reduced, and the entire network became less integrated (Table S2).

Within the fungal community, EcM fungal network was more connected than non‐EcM fungal network (Figure S6). This may be related to the predominance of physical EcM networks (in the more traditional sense) in timberline ecosystems [26]. The higher connectedness within the EcM fungal community network may facilitate the colonization and spread of *B. ermanii* population at the timberline. In addition, the differences between EcM and non‐EcM fungal effects on the multi‐kingdom network (including topological properties and stability) were not obvious (Table S6, Figure S6). The addition of EcM and non‐EcM fungi individually enhanced the network stability and connectivity of plants, nematodes, and archaea (Figure S4); however, the enhancement was weaker than the effect of the entire fungal community. Consequently, the fungal community should be considered as a whole in either association network studies or when formulating conservation strategies, which may be different from the widespread approach in classical fungal community ecology studies [38, 50].

In addition to the multi‐kingdom network results, MRM and SEM together showed that the plant community strongly affected the fungal community, whereas the fungal community significantly affected the archaeal and nematode community, and vice versa (Figure 6B, Table S8). Fungi had no direct effect on plants, which is similar to our previous finding in alpine grasslands [38]. In addition, there were no direct effects between fungi and bacteria, which is inconsistent with the finding of a global soil microbiome survey [51]. Nevertheless, soil fungi indirectly affected soil bacteria via their effect on nematodes and archaea (Figure 6B), and exhibited the largest proportion of cross‐kingdom links with bacteria (Figure 5). Overall, the results reflect that the fungal community is at the center of multi‐kingdom community interactions and is the ‘bridge’ linking aboveground and belowground communities in our study area. For example, the plant community mediates EcM fungal community and biomass by rhizodeposits, and the variation of the fungal community will further affect the nematode community by decreasing the abundance of fungivorous nematodes [52].

Last but not least, it should be noted that our inferred multi‐kingdom network stems from statistical associations rather than directly verified biotic interactions, although the multi‐kingdom network analyses are advancing our understanding of the actual roles of species belonging to different kingdoms in a multi‐kingdom community. It remains an open question as to how well multi‐kingdom networks represent real biotic interactions [53, 54]. Using machine learning algorithms and trait‐based prediction is one of important research interests in the future [55]. In addition, experimental validation is also an effective way to improve the reliability of network analyses, despite the limited interactive species and small habitat range [19, 40].

## CONCLUSION

At the network and community levels, we demonstrated that (1) soil fungi, including EcM, AMF, saprotrophic, and other trophic fungi together play the role of “broker” in the multi‐kingdom network. When fungi are integrated into the networks of plants, archaea, nematodes, and bacteria, they increase their connectivity and stability while decreasing their modularity. In particular, fungi are inclined to build cross‐kingdom edges and module‐to‐module connections. (2) Soil fungal communities play the role of a “bridge” in the multi‐kingdom community. Fungal communities are located at the center of the multi‐kingdom community, and they link plants and soil biota (Figure 6). The findings in the present study highlight the roles of fungal communities and their interactions (particularly EcM fungi [56]) with other biota. The findings, which enhance our understanding of the potential roles and interactions of different soil biota and plants, could facilitate the formulation of appropriate conservation strategies in high elevation timberline ecosystems, and in other similar ecosystems globally.

## METHODS

### Study area, sample collection, and plant surveys

The study area is located on the northern slope of Changbai Mountain, Northeast China. In the area, *B. ermanii* grows over the elevational range of about 1700–2100 m a.s.l., forming a broad timberline ecotone from closed forests to alpine tundra in the mountain top [57, 58]. Along the ecotone, the soils neighboring 30 mature *B. ermanii* individuals were sampled along the elevation from 1688 to 2113 m (Figure S9A). With the trunk as the center and the diameter at breast height (DBH) as the distance from the stem, four soil cores (diameter = 3.5 cm, depth = 10 cm) were collected after the removal of litter and mixed as a composite soil sample (Figure S9B). In addition, plant community composition and plant cover, including trees, shrubs, and herbs, were recorded in the plots near each of the *B. ermanii* individuals (Figure S9B). In the present study, plant communities in the periphery of *B. ermanii* individuals changed significantly along the elevation (*R* ^2^ = 0.321; Figure S10), and plant richness showed a U‐shaped curve, with the lowest richness in the mid‐elevation (Figure S11).

The following parameters associated with the focal *B. ermanii* individuals were recorded: population density (number of *B. ermanii* individuals in the tree survey plots), litter depth, tree height, canopy diameter, DBH, and distance to the forest edge. Root C, root N, root phosphorus (P), root potassium (K), root calcium (Ca), root magnesium (Mg), root manganese (Mn), root aluminum (Al), root iron (Fe), root C/N ratio, root N/P ratio, as well as the lignin, cellulose, hemicellulose, sugar, protein, free amino acid and free fatty acid contents in roots were determined from the root samples. Soil moisture, pH, conductivity, nitrate N, ammonium N, dissolved organic carbon (DOC), dissolved organic nitrogen (DON), total C, total N, total P, C/N ratio, N/P ratio, total K, total Ca, total Mg, total Mn, total Al, total Fe, available P, available K, available Ca, available Mg, available Mn, available Al, available Fe, as well as the proportions of clay, silt and sand were measured from the soil samples. The protocols have been described in our previous study [34] and the data are summarized in Figures S12 and S13.

### Molecular analyses

The modified Baermann funnel method [59] was used to enrich and extract nematode communities. Based on the motility of nematodes, 50‐g screened (2 mm) soil was immersed in 50‐ml sterilized water and incubated for 48 h at approximately 25°C. The soil leaching liquid (including living nematodes) was collected in a 50 ml centrifugal tube. For every sample, two tubes of nematode liquids were collected. After centrifugation at 9000*g* for 20 min, two nematode pellets were combined into one composite sample and frozen for DNA extraction. Screened soil (0.5 g) was used to extract fungal, bacterial, and archaeal DNA. The FastDNA Spin kit for Soil (MP Biomedicals) was used to extract all soil biota DNA.

The 18S small subunit (SSU) ribosomal gene was amplified using the primers NF‐1 (GGTGGTGCATGGCCGTTCTTAGTT) and 18Sr2b (TACAAAGGGCAGGGACGTAAT) for the nematode community [60]. The internal transcribed spacer 1 (ITS1) ribosomal gene was amplified using the primers ITS1‐F (CTTGGTCATTTAGAGGAAGTAA) and ITS2 (GCTGCGTTCTTCATCGATGC) for the total fungal community [34]. To obtain much more information on the phylum Glomeromycota (fungi), we also performed a two‐step PCR (first round: AML1 (ATCAACTTTCGATGGTAGGATAGA) and AML2 (GAACCCAAACACTTTGGTTTCC); second round: AMV4.5NF (AAGCTCGTAGTTGAATTTCG) and AMDGR (CCCAACTATCCCTATTAATCAT)) to amplify the 18S rRNA gene [61]. The 16 S rRNA gene was amplified using the primers 515 F (5’‐GTGCCAGCMGCCGCGG‐3’) and 907 R (5’‐CCGTCAATTCMTTTRAGTTT‐3’) for bacteria [62]. The primers 524F10extF (TGYCAGCCGCCGCGGTAA) and Arch958RmodR (YCCGGCGTTGAVTCCAATT) were used to amplify archaea [63]. All the PCR products were normalized to equimolar amounts and sequenced on an Illumina MiSeq PE300 platform (Majorbio Company).

Raw sequences were processed using the ASV method in the Quantitative Insight into Microbial Ecology 2 (QIIME2) pipeline [64]. The raw sequences with average quality scores of <20 or read lengths of <80 bp were filtered using Trimmomatic [65] and merged using FLASH software [66]. The sequences were denoised using the DADA2 algorithm, and the ASVs were generated [67]. The SILVA SSU 138 release served as the reference database for nematode, bacterial, and archaeal taxonomy (https://www.arb-silva.de/) [68]. Before taxonomic assignments, the SILVA SSU 138 release was trained using the q2‐feature‐classifier (Pre‐fitted sklearn‐based taxonomy classifier [69]) with distinct primers for nematodes, bacteria, and archaea. The ASVs for AMF were assigned using BLAST against MaarjAM online (http://maarjam.botany.ut.ee/). The Unite v8.0 (http://unite.ut.ee) release for QIIME served as the reference database for fungal taxonomy [70]. Before assignment, the Unite release was also trained using the q2‐feature‐classifier with the ITS1‐F/ITS2 primers.

After removing the nontarget ASVs, 77,955 nematode reads, 1,184,347 fungal reads, 562,808 AMF reads, 1,486,828 bacterial reads, and 895,426 archaeal reads were retained, which corresponded to 246, 2,081, 182, 10,165, and 333 ASVs, respectively. After subsampling to the minimum reads per sample and merging the fungal and AMF ASV tables, four ASV tables for nematodes, fungi, bacteria, and archaea were retained (Table S9). Fungal functional guilds were assigned using FUNGuild [71].

### Statistical analyses

All statistical analyses were conducted in R v4.1.0 [72] and AMOS 21.0 (AMOS IBM). First, linear, quadratic, and cubic regression models were used to determine the variations in neighboring vegetation (e.g., richness and cover), *B. ermanii* associated factors, and soil properties along the elevation gradient. The model with the lowest Akaike's information criterion (AIC) value was selected (Tables S10–S12). Significant variation in neighboring plant community was tested using Permutational Multivariate Analysis of Variance in the vegan package [73]. After removing the highly collinear variables (*r* > 0.7), 40 variables were retained, which included seven neighboring floristic variables, 15 *B. ermanii*‐associated factors, and 18 soil properties (Figure S8). Subsequent analyses were based on the 40 variables above.

The multi‐kingdom networks of plants, nematodes, fungi, bacteria, and archaea were constructed using the extended SPIEC‐EASI method [19]. In addition, the single‐kingdom networks of plants, nematode, fungi, bacteria, and archaea were constructed, separately, using the SPIEC‐EASI method [74]. SPIEC‐EASI is robust against community compositionality bias [12, 74]. ASVs occurring in <5 samples were removed from network analyses (Table S9). Plant species table (absence/presence) including 102 species was also used for network analyses. Network properties, including node number, edge number, mean connectivity, modularity, average path length, and proportions of negative and positive edges were selected for use in comparison of networks. Node number, edge number, mean connectivity, modularity, and average path length were calculated using the igraph package [75]. The proportions of negative and positive edges were calculated using Gephi v0.9.2 [76]. All network diagrams were visualized using Gephi v0.9.2. In the entire multi‐kingdom network, node properties, including degree and betweenness, were also calculated using the igraph package [75]. To compare degree and betweenness among kingdoms, the node degree and betweenness were transformed according to the method of de Vries et al. [20], and their differences were tested using the *kruskalmc* function in the pgirmess package [77]. In addition, the nodes from plants, nematodes, fungi, bacteria, and archaea were assigned to the peripheral, connector, module hub, or network hub, according to their patterns of within‐ and between‐module connections [13]. In addition, network stability was estimated by removing 50% of nodes in a stepwise fashion to assess how rapidly the natural connectivity degraded in different single‐kingdom networks as well as in the multi‐kingdom networks in the absence or presence of fungi, nematodes, bacteria, and archaea [78]. The lower the absolute value of the slope, the more stable the network [79]. To compare the roles of EcM and non‐EcM fungi in association networks, the aforementioned network analyses were also performed for EcM and non‐EcM fungi, separately.

We analyzed the levels of preference of fungal links for plants and other soil biota in the entire multi‐kingdom network. Based on the node numbers of plants and other soil biota, the expected proportions of fungal links were first calculated. Subsequently, the deviates between the observed proportions of fungal links and the expected proportions were calculated to represent the preference for fungal links. In addition, 999 random networks with the same numbers of nodes and edges (incl. positive: negative edge proportions) were generated for the fungal single‐kingdom network and fungal within‐kingdom network based on the Erdos‐Reyni model [80]. Afterward, the negative edge proportions were calculated in the links of saprotrophic and EcM fungi as well as in the remaining links within fungi. To enable comparisons across networks and trophic groups, the negative edge proportions were *Z*‐score normalized, which accounted for the variation in species richness and number of observed links [81]. The *Z*‐scores of negative edge proportion were defined as *Z* = (*V*_observed_ − *A*_randomized_)/SD_randomized_, where *V*_observed_ is the observed value, and *A*_randomized_ and SD_randomized_ are the average and standard deviation of the 999 randomized matrices.

To test the biotic interactions among plants, nematodes, fungi, bacteria, and archaea at the community level, two MRM models (i.e., MRM #1 and MRM #2) were constructed [82, 83]. In MRM #1, the 40 noncollinear variables, geographic distance, and neighboring plant community were treated as the predictors for the compositions of microbial and nematode communities. In MRM #2, the community compositions of soil nematodes, fungi, bacteria, and archaea were also added as predictors of community compositions in each kingdom, separately. By comparing the explanatory rates of the two MRM models, we determined whether biotic interactions among soil biota affected their respective community compositions. Finally, we constructed the expected SEM model based on two hypotheses: (1) plant community affects soil biota (incl. fungi) by modifying soil properties; (2) fungi community affects plants and soil biota (excluding fungi), and vice versa. Then we obtained the optimal model based on AIC and other statistical parameters in AMOS, which showed the community–to–community relationships on the whole.

## AUTHOR CONTRIBUTIONS

Haiyan Chu and Teng Yang conceived and designed the study. Teng Yang, Xu Liu, Gui‐Feng Gao, and Ke Dong performed the experiments and analyzed the data. Teng Yang, Leho Tedersoo, Jonathan M. Adams, and Haiyan Chu wrote the manuscript and advised on the interpretation of the results. All the authors read and approved the final manuscript.

## CONFLICT OF INTEREST

The authors declare no conflict of interest.

## Supporting information

Supporting information.

Supporting information.

## Data Availability

The datasets supporting the conclusions of this article are available in the Genome Sequence Archive (GSA) under BioProject accession number PRJCA010746 [84]. Background data, including neighboring plant community, plant cover, *B. ermanii*‐associated factors, and soil properties, can be shared by the corresponding author upon reasonable request. Supplementary materials (figures, tables, scripts, graphical abstract, slides, videos, Chinese translated version, and update materials) may be found in the online DOI or iMeta Science http://www.imeta.science/.
