# Supplementary material for: Fungi stabilize multi‐kingdom community in a high elevation timberline ecosystem
Source: Imeta. 2022 Aug 15;1(4):e49. doi: 10.1002/imt2.49 (PMC10989762; doi:10.1002/imt2.49)
Supplement: Supplementary file 1 — Supporting information. [file IMT2-1-e49-s001.docx]

Supporting information for

**Fungi stabilize multi-kingdom community in a high elevation timberline ecosystem**

**Author Names:** Teng Yang^1,5^, Leho Tedersoo^2^, Xu Liu^1,5^, Gui-Feng Gao^1,5^, Ke Dong^3^, Jonathan M. Adams^4^, Haiyan Chu^1,5*^

^1^State Key Laboratory of Soil and Sustainable Agriculture, Institute of Soil Science, Chinese Academy of Sciences, East Beijing Road 71, Nanjing 210008, China

^2^Mycology and Microbiology Center, University of Tartu, 2 Liivi, Tartu 50409, Estonia

^3^Life Science Major, Kyonggi University, Suwon 16227, South Korea

^4^School of Geographic and Oceanographic Sciences, Nanjing University, Nanjing 210008, China

^5^University of Chinese Academy of Sciences, Beijing 100049, China

***Corresponding author:** Haiyan Chu

Tel: +86 02586881356, E-mail: [hychu@issas.ac.cn](mailto:hychu@issas.ac.cn)

**
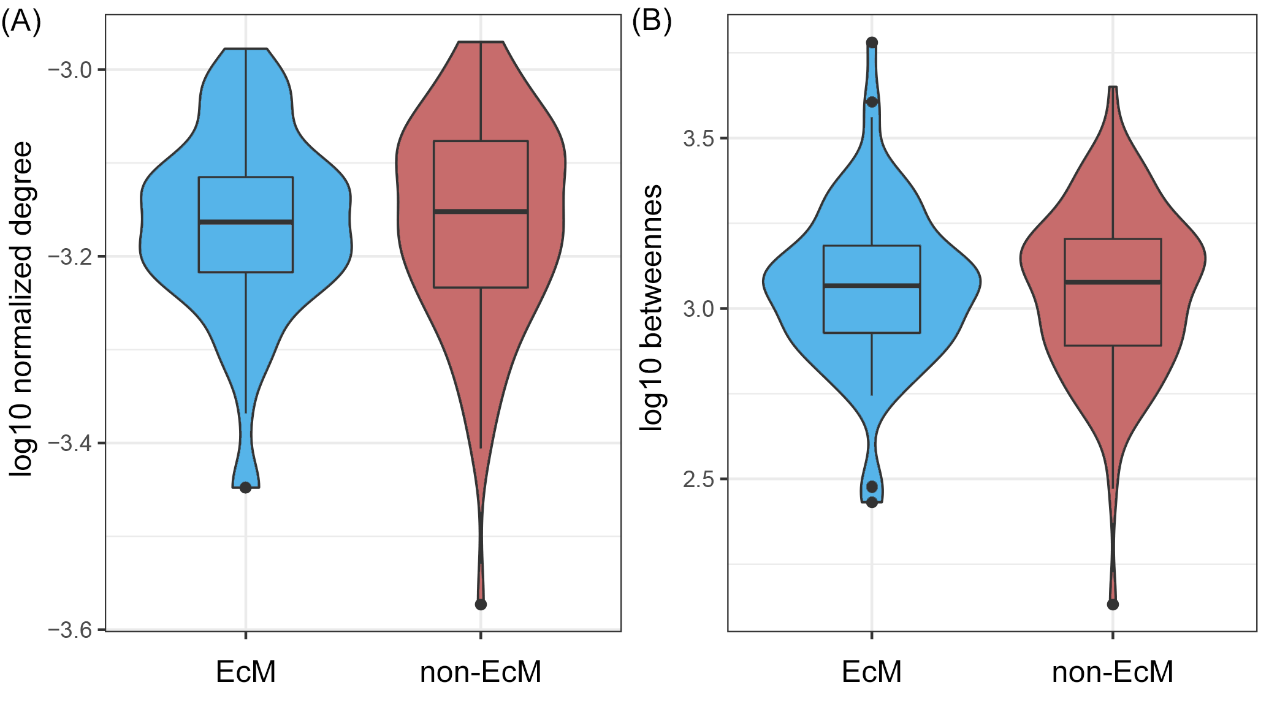
Figure S1** Node connectedness and centrality of ectomycorrhizal (EcM) and non-EcM fungi in the multi-kingdom network. (**A**) Node connectedness is represented by log10 normalized degree, and (**B**) centrality is represented by log10 betweenness. There were no significant differences in node connectedness and centrality between EcM and non-EcM fungi.


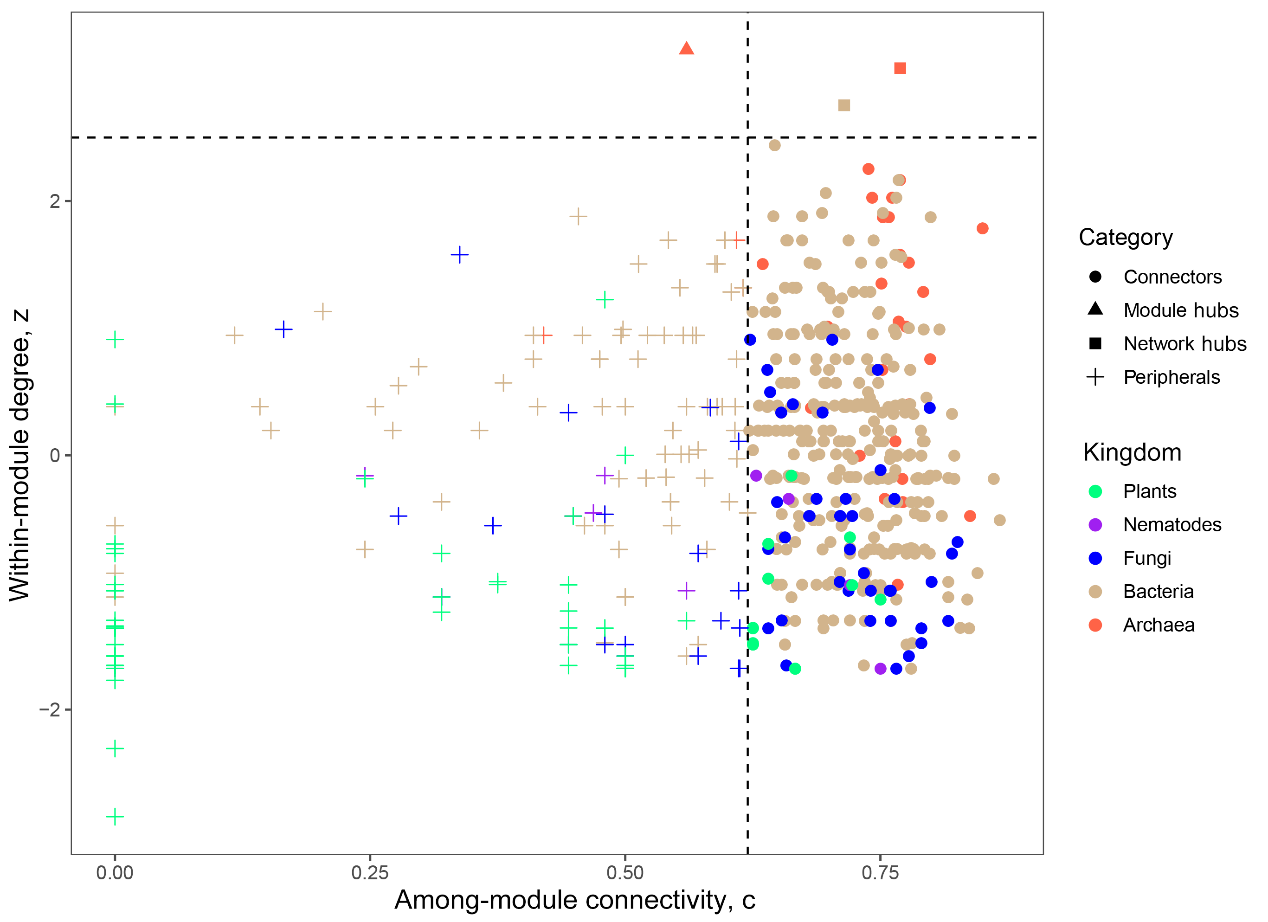
**Figure S2** *Z*-*c* plot showing the topological roles of plant, nematode, fungal, bacterial, and archaeal nodes in the multi-kingdom network. Different shapes represent the topological roles in the entire association network, and different colors represent the kingdoms. Dashed lines indicate the critical values according to Olesen et al. (2007).

**
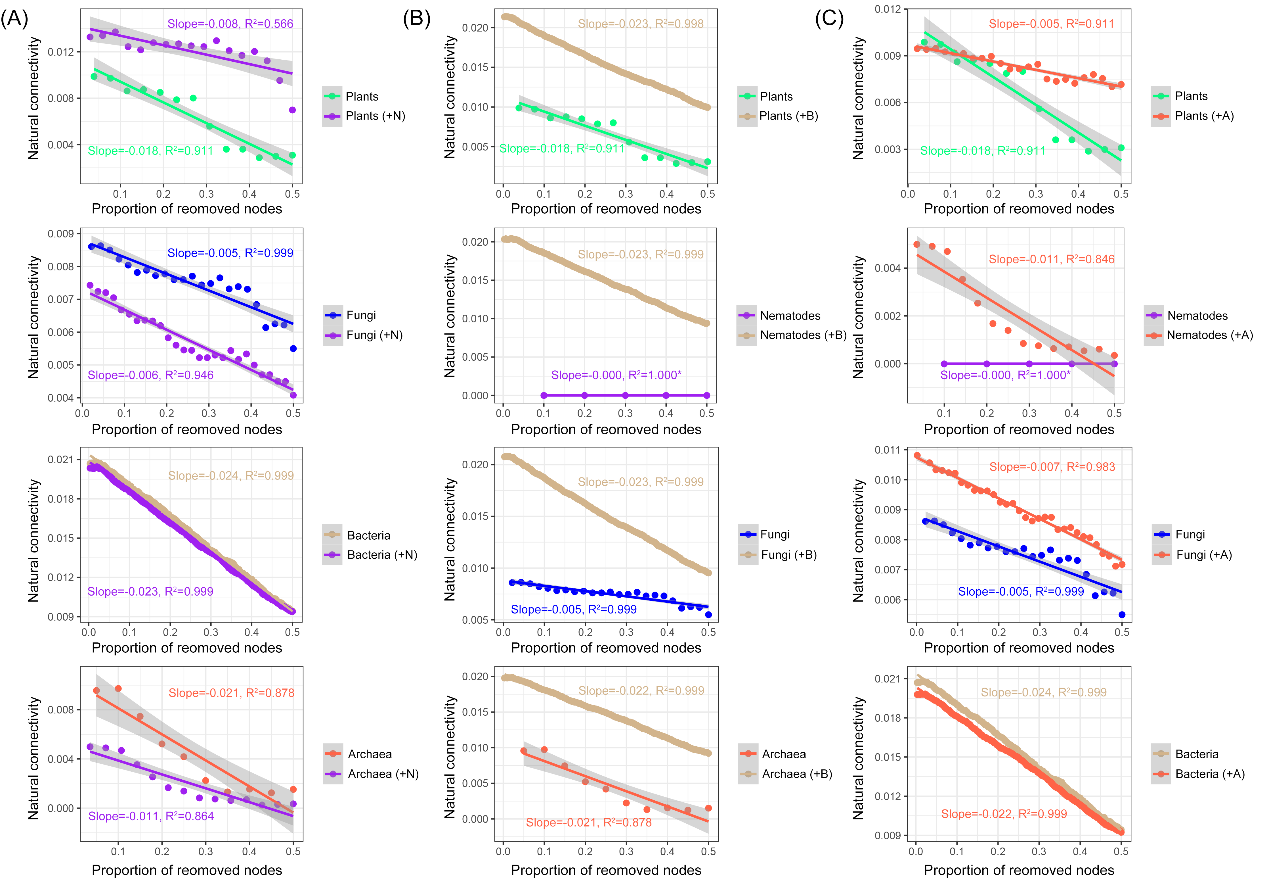
Figure S3** Changes in network stability with the addition of nematodes (**A**), bacterial (**B**), and archaeal (**C**) interactions. Decreasing natural connectivity trend is fitted with 50% nodes lost, and the R square and slope are shown in diagrams. “+N”, “+B” and “+A” represent the addition of nematode, bacterial, and archaeal interactions, respectively, based on single-kingdom networks.

**
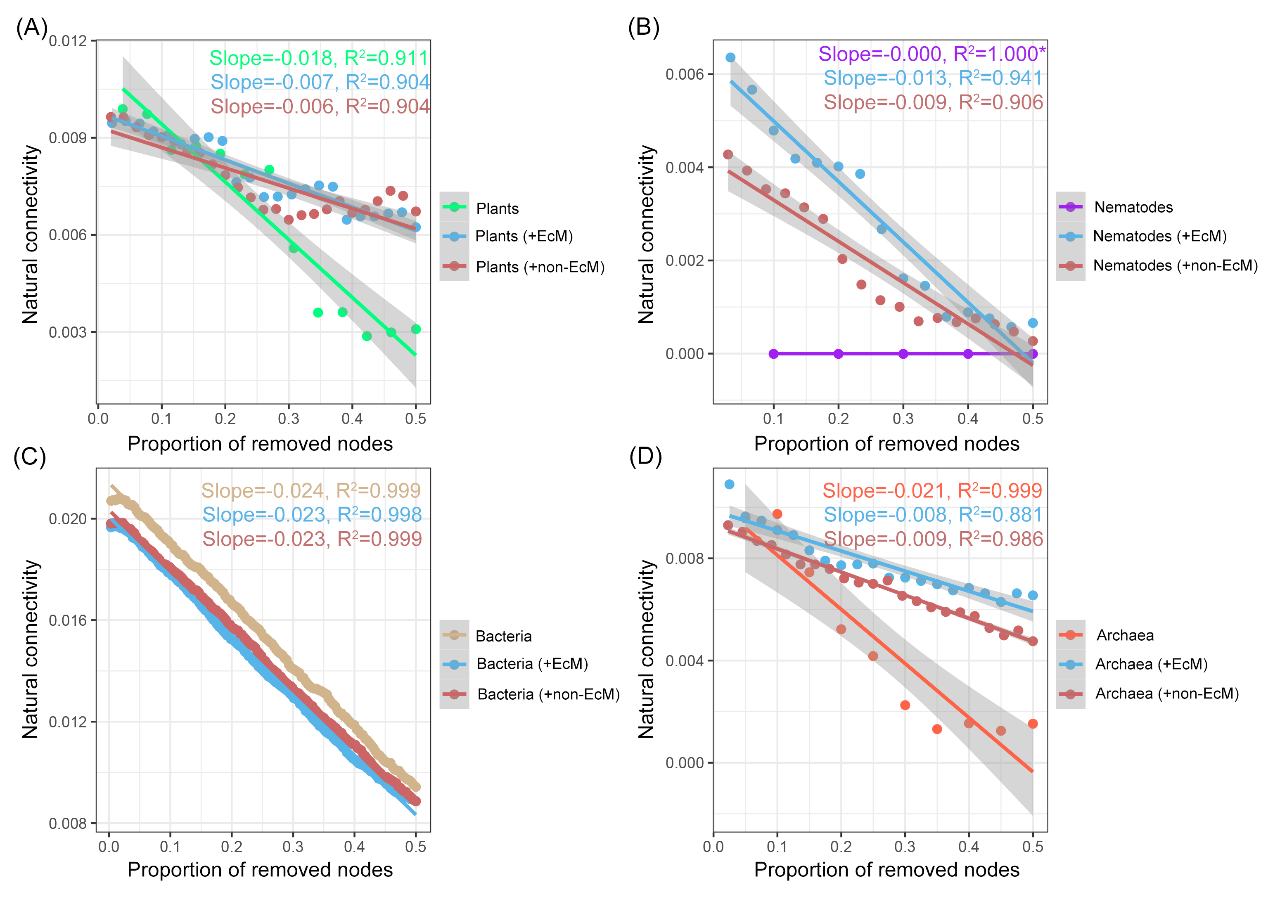
Figure S4** Effects of ectomycorrhizal (EcM) and non-EcM fungi on network stability of plants (**A**), nematodes (**B**), bacteria (**C**), and archaea (**D**). Decreasing natural connectivity trend is fitted with 50% nodes lost, and R square and slope are shown in diagrams. “+EcM” and “+non-EcM” represent the addition of EcM fungi and non-EcM fungi, respectively, based on single-kingdom networks.


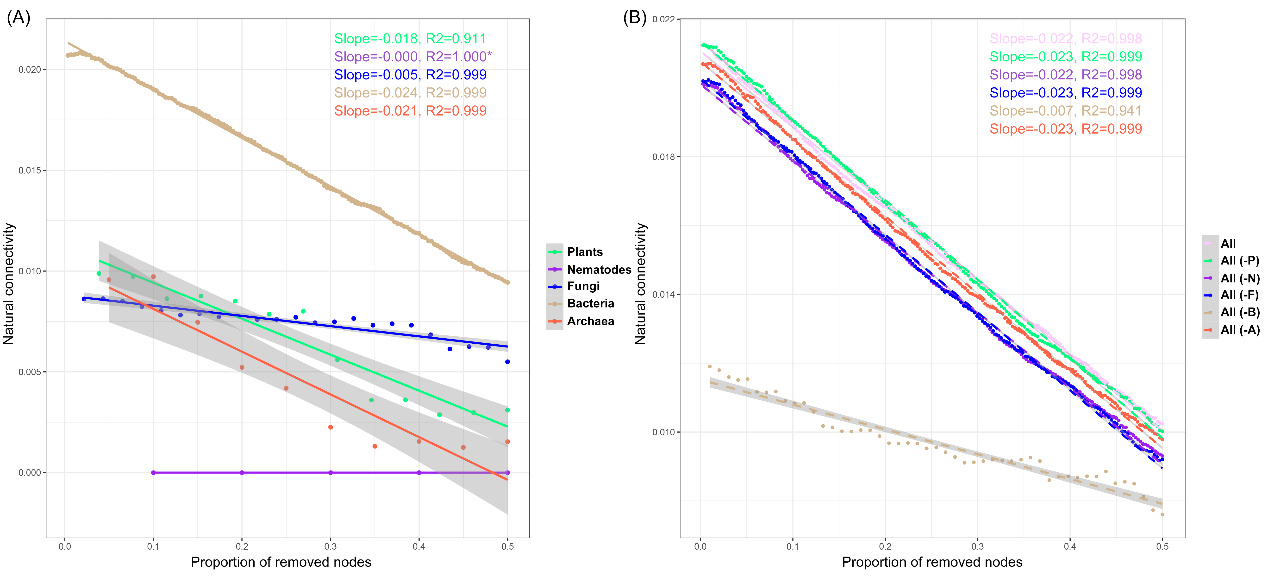
**Figure S5** Network stability of single-kingdoms (**A**) and multi-kingdom (**B**). (**A**) *nematodes cannot form single-kingdom networks by themselves; therefore the degradation rate of natural connectivity is zero in the nematode network. (**B**) All (-P) represents the exclusion of plant interactions from the entire multi-kingdom network. All (-N) represents the exclusion of nematode interactions from the entire multi-kingdom network. All (-F) represents the exclusion of fungal interactions from the entire multi-kingdom network. All (-B) represents the exclusion of bacterial interactions from the entire multi-kingdom network. All (-A) represents the exclusion of archaeal interactions from the entire multi-kingdom network. Decreasing natural connectivity trend is fitted with 50% nodes lost, and the R square and slope are shown in diagrams.

**
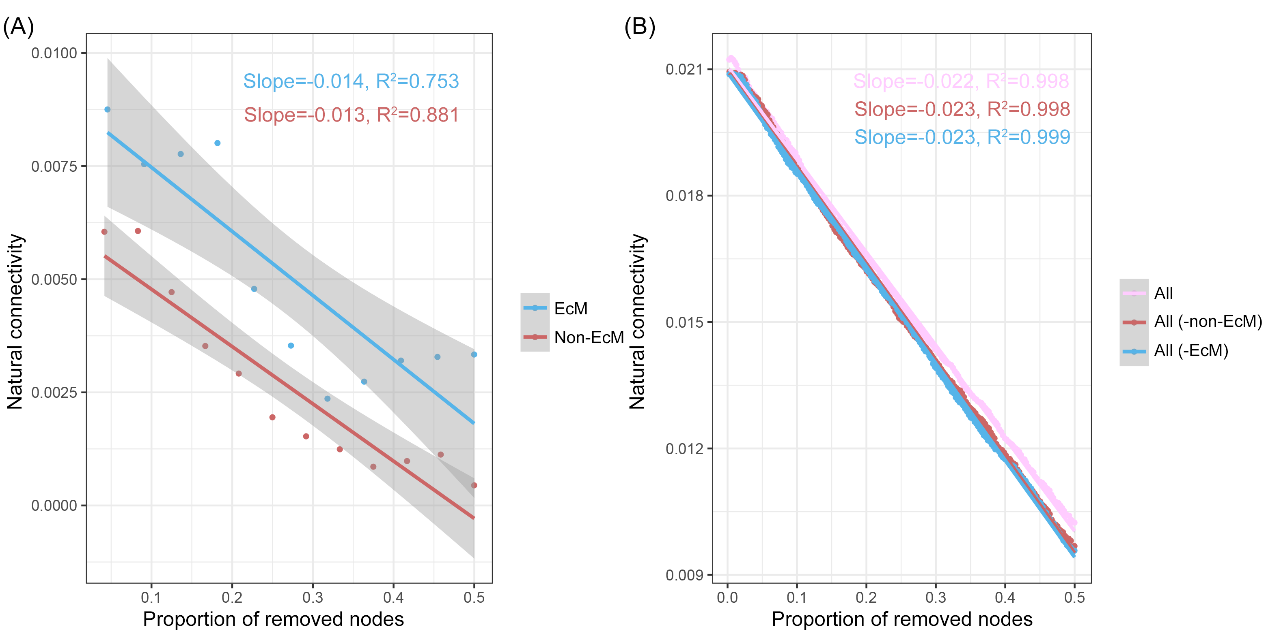
Figure S6** Network stability of ectomycorrhizal (EcM) and non-EcM fungi (**A**) and the entire multi-kingdom following EcM and non-EcM fungi removal (**B**). The decreasing trend of natural connectivity is fitted with 50% nodes lost, and R square and slope are shown in diagrams. All (-EcM) represents exclusion of EcM fungal interactions from the entire multi-kingdom network, and all (-non-EcM) represents the exclusion of non-EcM fungal interactions from the entire multi-kingdom network.

**
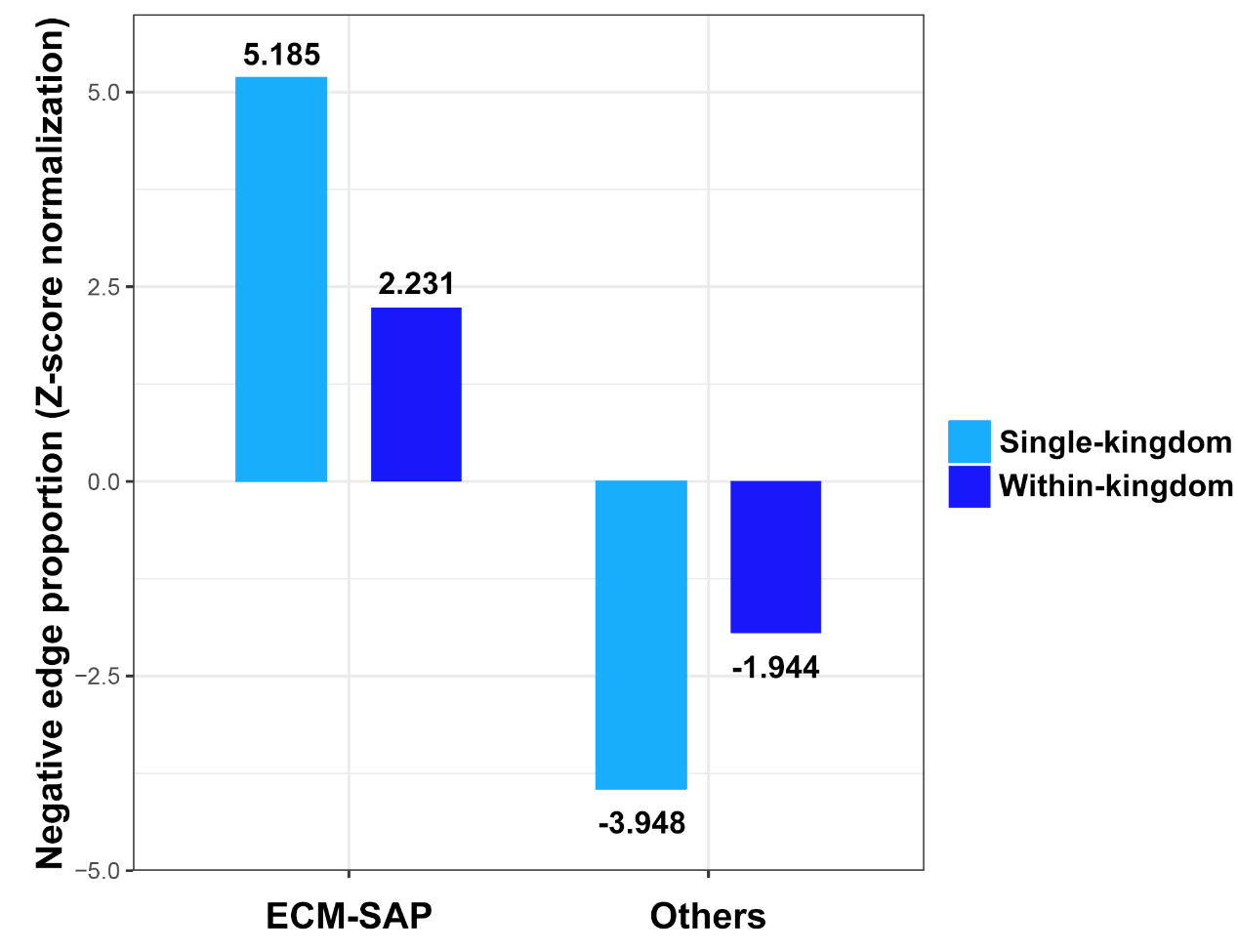
Figure S7** Negative edge proportions in the single-kingdom and within-kingdom fungal networks. Here, the negative edge proportions are standardized by Z-score normalization (see Methods). Single-kingdom means fungal single-kingdom network, while Within-kingdom means fungal within-kingdom network that is a subset of the multi-kingdom network. “ECM-SAP” means the links between saprotrophic and ectomycorrhizal fungi, and “Others” means the remaining links in the fungal kingdom.


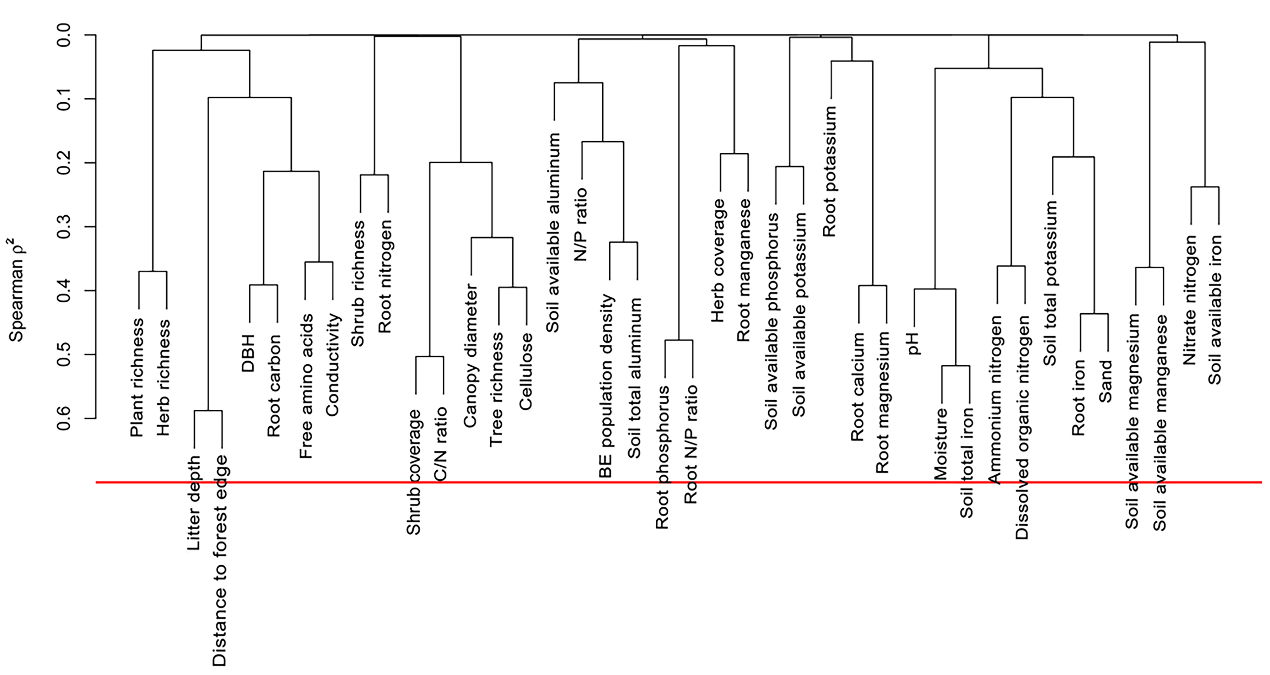
**Figure S8** Correlations among the 40 variables after removal of the highly collinear variables. The red line represents a value of 0.7, which means that the remaining 40 variables are not highly colinear.


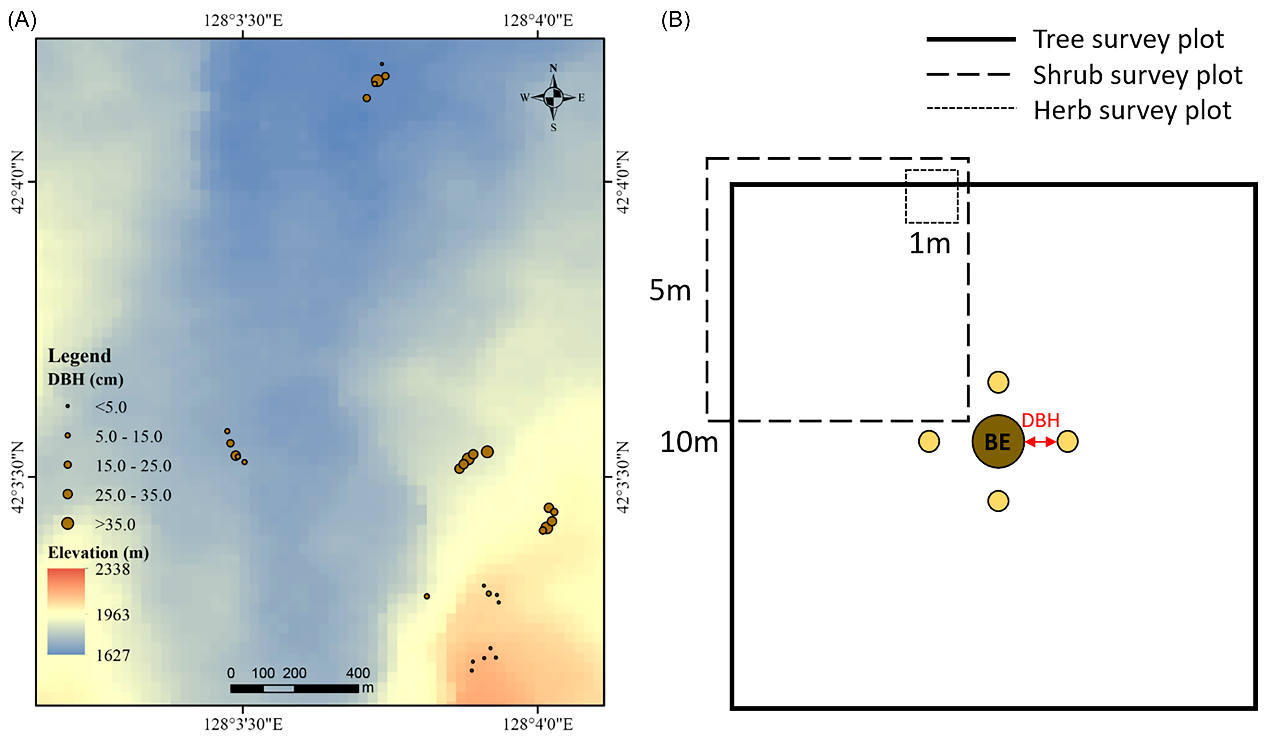
**Figure S9** Sampling maps at the landscape (**A**) and plot (**B**) scales. **A**: Each brown point represents a *Betula ermanii* individual, and node sizes indicate the diameter at breast height (DBH). Elevation is indicated by the color gradient. The whole area is located in the core region of Changbai Nature Reserve. N = 30. **B**: BE means a *B. ermanii* individual around which four soil cores (yellow points) with depths of 0–10 cm and a diameter of 3.5 cm are sampled. The litter depths are also measured in the four points. Meanwhile, plant survey plots are set randomly in one of the four corners. The population densities of *Betula ermanii* are recorded in the same tree survey plots.


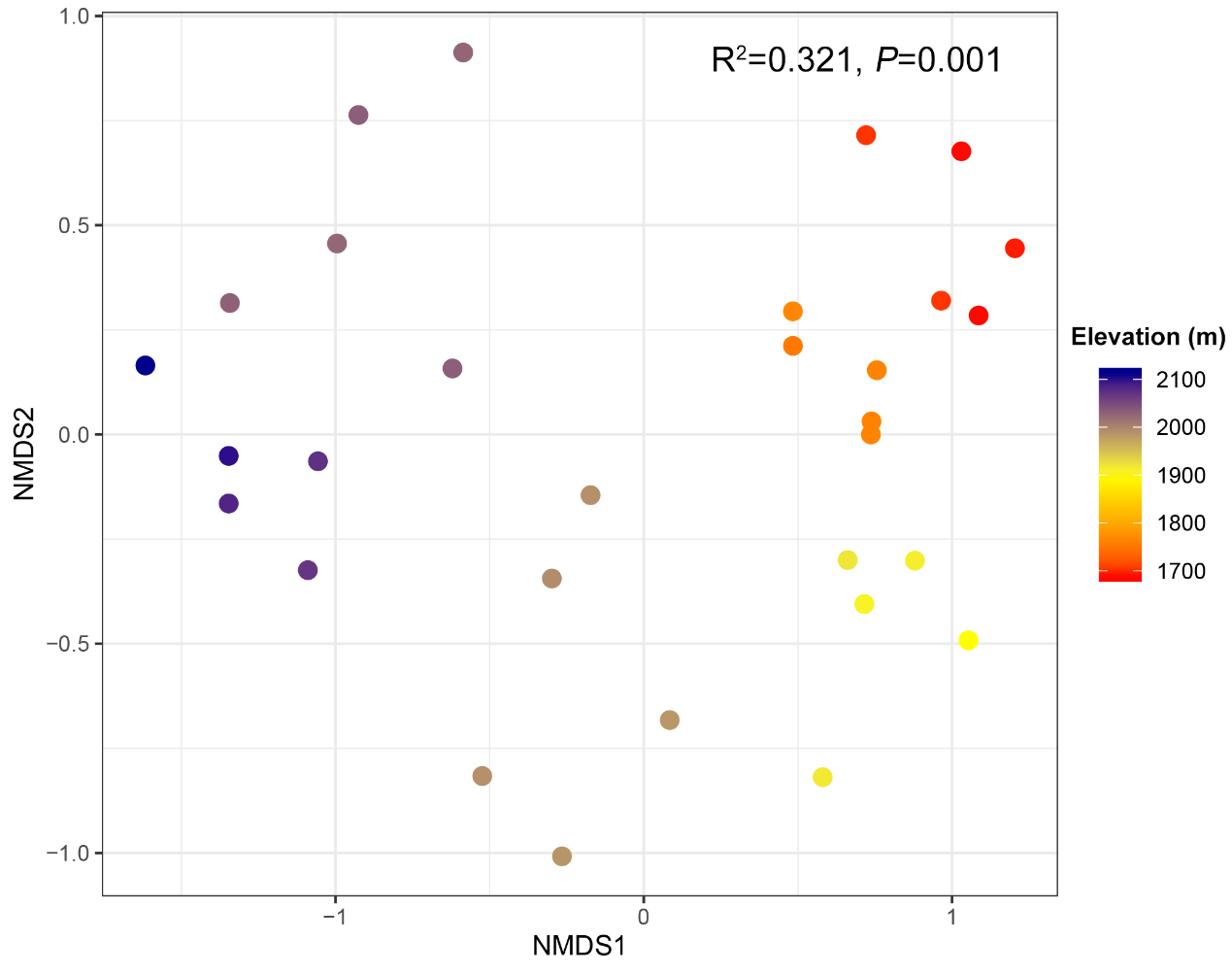
**Figure S10** Two-dimensional ordination using Nonmetric Multidimensional Scaling (NMDS), based on Jaccard dissimilarity index. Each point (30) represents the neighboring plant community of an individual *Betula ermanii* tree.

**
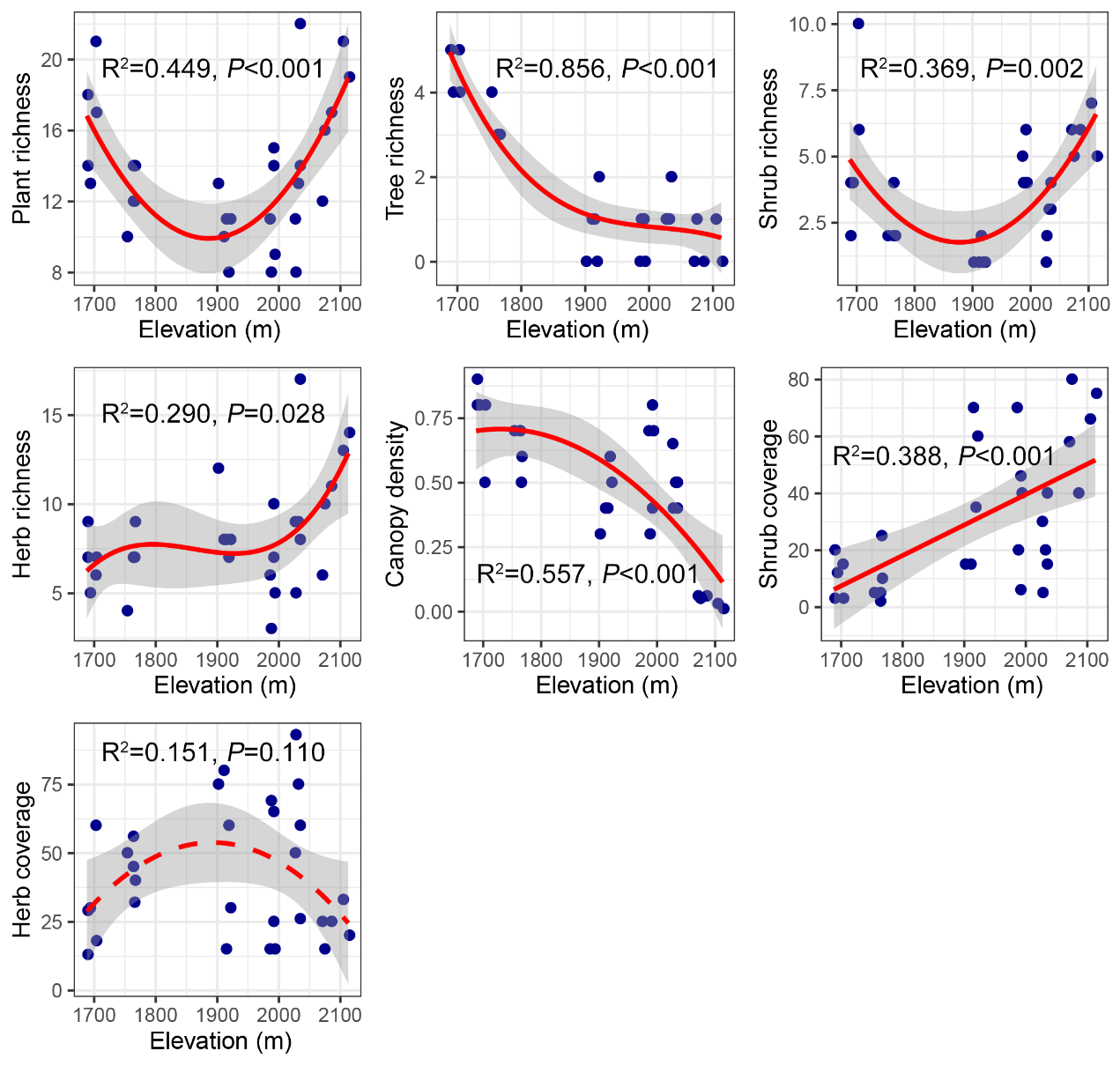
Figure S11** Variations in neighboring floristic richness and plant cover along the elevation gradient. Linear, quadratic and cubic models were fitted, and the optimal models were selected based on Akaike Information Criterion (AIC) values (Table S10). The solid red lines indicate significant differences in the relationships, while the dashed lines indicate no significant difference in the relationships. N = 30.


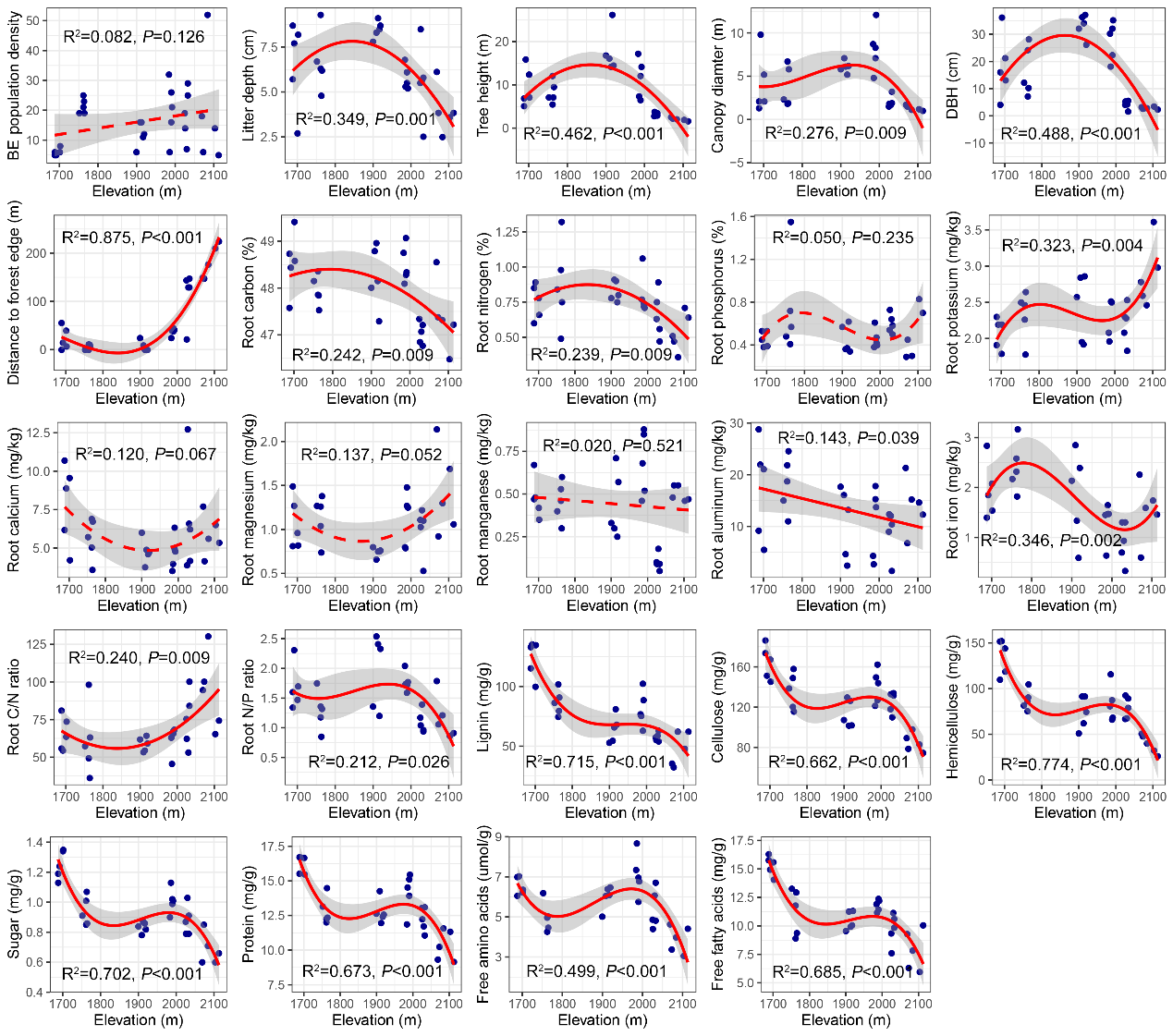
**Figure S12** Variations in *Betula ermanii*-associated factors along the elevation gradient. Linear, quadratic, and cubic models were fitted, and the optimal models were selected based on Akaike Information Criterion (AIC) values (Table S11). The solid red lines indicate significant differences in the relationships, whereas the dashed lines indicate no significant differences in the relationships. N = 30.


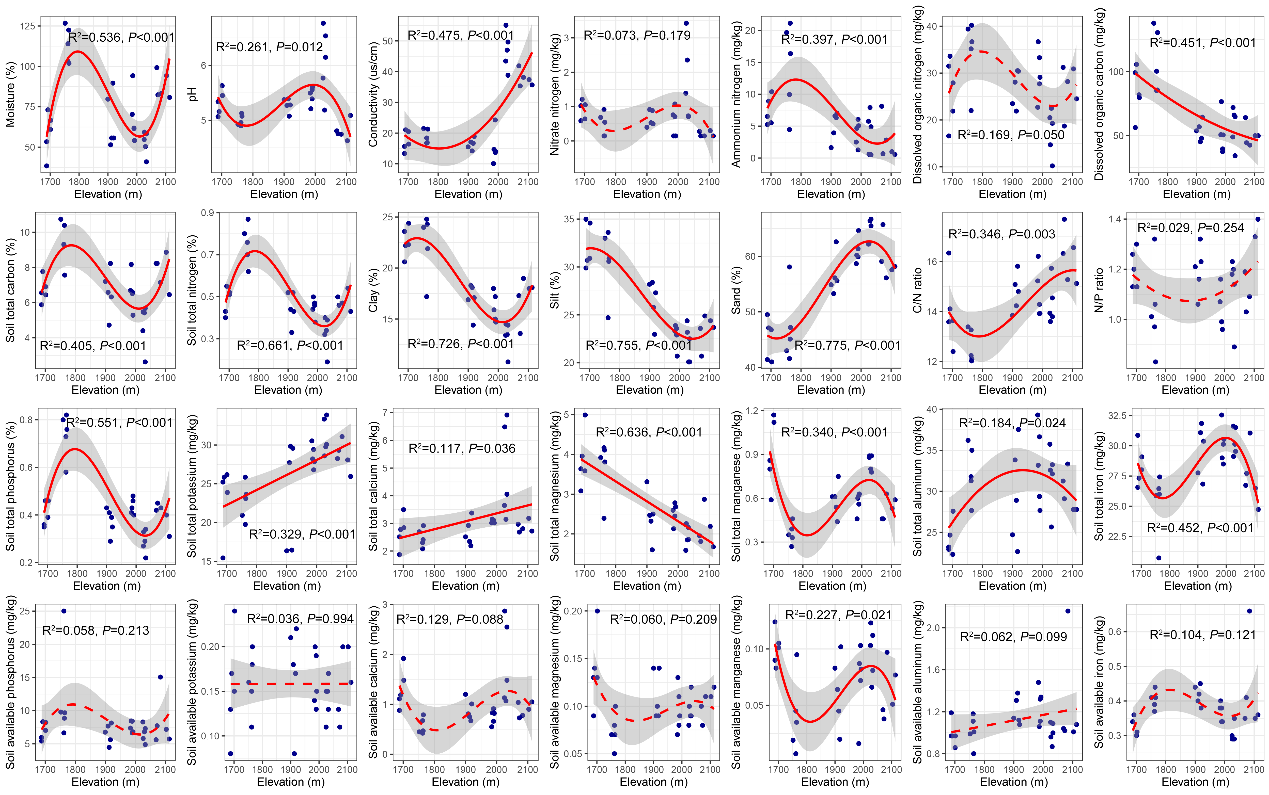
**Figure S13** Variations in soil properties along the elevation gradient. Linear, quadratic, and cubic models were fitted, and the optimal models were selected based on Akaike Information Criterion (AIC) values (Table S12). The solid red lines indicate significant difference in the relationships, whereas the dashed lines indicate no significant difference in the relationships. n=30.
